# Stability, Aromaticity, and Photophysical Behaviors of Macrocyclic Molecules: A Theoretical Analysis

**DOI:** 10.3389/fchem.2020.00776

**Published:** 2020-09-04

**Authors:** Wei Wei, Wenhui Ren, Wei Jian, Baohui Xia, Hongxing Zhang, Fu-Quan Bai, Wei Li

**Affiliations:** ^1^Laboratory of Theoretical and Computational Chemistry, Institute of Theoretical Chemistry, Jilin University, Changchun, China; ^2^School of Chemistry and Materials Science, Hunan Agricultural University, Changsha, China; ^3^College of Chemistry, Jilin University, Changchun, China

**Keywords:** porphyrin, DFT/TD-DTF, aromaticity, macrocyclic molecules, molecular modification

## Abstract

The macrocyclic molecules with terthiophene (TTH) isomers unit exhibit intriguing properties in terms of aromaticity, stability, and absorption. In this work, we theoretically designed a series of macrocyclic molecules featured with TTH and dithienothiophene (DTT) π-conjugated building units, which are used to permute pyrrole unit in porphyrin skeleton. Density functional theory and time-dependent DFT methods are used to evaluate the performance of the designed molecules. Our simulations show that molecules **1**–**3** exhibit excellent optoelectronic performance. Specifically, the molecule with the DTT unit is more stable than the one with TTH unit in terms of aromaticity and aromatic stabilization energy. This is because DTT unit enhances the coplanarity of the molecular material, facilitating electronic communication. Calculation of vertical electronic excitations suggests the absorption feature of these molecules is mainly contributed by the electronic excitations of higher occupied molecular orbital (HOMO) → lowest unoccupied molecular orbital (LUMO)+1 and HOMO-1 → LUMO. Judging from the key parameters determining the overall performance, **3** stands out because of its good planarity, large HOMO–LUMO gap, and strong aromaticity among all molecules. Interestingly, molecule **1** has the current density flow distributes around the outer section of TTH unit; in contrast, molecule **3** with DTT unit has the current density flow located at the inner section of DTT, which is beneficial for stability and aromaticity. Second-order perturbation energies are calculated to rationalize this observation. We expect that these research results can provide valuable insights into the rational design of novel molecular materials for a variety of applications.

## Introduction

Porphyrins, the tetrapyrrolic macrocycles with 18 π-electrons, have attracted the attention of chemists for a long time in view of their diverse applications such as material science and medicine (Drain et al., 2009; Barona-Castaño et al., 2016; Bryden and Boyle, 2016; Tang et al., 2019). Porphyrins feature the highly conjugated macrocycles composed of four modified pyrrole subunits interconnected at α carbon atoms via methine bridges. Sapphyrins are an important group of expanded porphyrin with 22 π-electrons that show anion binding characteristics (Richter and Lash, 2004). Since the first synthesis by Johnson et al. (Broadhurst et al., 1972) sapphyrins have been well-established in recent years because of the availability of easy and efficient synthetic methods (Chatterjee et al., 2017).

Carbasapphyrin (Richter and Lash, 1998), benzosapphyrin (Panda et al., 2005), and dithiabenzisapphyrin (Jeong et al., 2008) belong to the most popular sapphyrins series, and they are obtained by modifying the pyrrolic subunits. Carbasapphyrins feature a strong diatropic ring current due to the presence of 22 π-electrons delocalization pathways (Chatterjee et al., 2017). The optical absorption spectrum of dioxabenzosapphyrin exhibits both Q (600–800 nm) and Soret (approximately 400 nm) bands (Cho et al., 2008). Many of the modified sapphyrins show dramatically different canonical properties such as aromaticity, absorption features, and metal cation complexation behavior. It has been shown these properties have strong relation to the frontier orbital energy levels, which depends on fundamental structures. It is noteworthy that terthiophene (TTH) is usually used as the π-conjugated building unit to permute pyrrole unit in porphyrin skeleton (Moriaty et al., 1985). In addition, dithienothiophene (DTT) is an electron-rich rigid fragment that has been frequently used in electronic and optoelectronic materials (Frey et al., 2002). Therefore, it would be particularly interesting to develop novel macrocyclic molecules based on TTH and DTT subunits.

Center to the performance of macrocycle is aromaticity (Chen et al., 2019). Theoretically, various parameters such as geometric (Listunov et al., 2018), energetic (Rakhi and Suresh, 2016; Nowroozi and Rad, 2017), magnetic (Torrent-Sucarrat et al., 2017), and electronic properties (Poater et al., 2005) have been developed to evaluate aromaticity and antiaromaticity. Because of the simplicity and applicability, nucleus-independent chemical shift (NICS) (Schleyer et al., 1996) analysis was also developed to determine the magnetic properties of molecules. The NICS values can be calculated at the geometrical center of a ring or above a molecular plane. As for the stability of macrocycle, aromatic compounds are, generally, substantially more stable than antiaromatic compounds. The higher occupied molecular orbital (HOMO)–lowest unoccupied molecular orbital (LUMO) gap value is also indicative of the stability of macrocycle molecule.

**Scheme 1:**
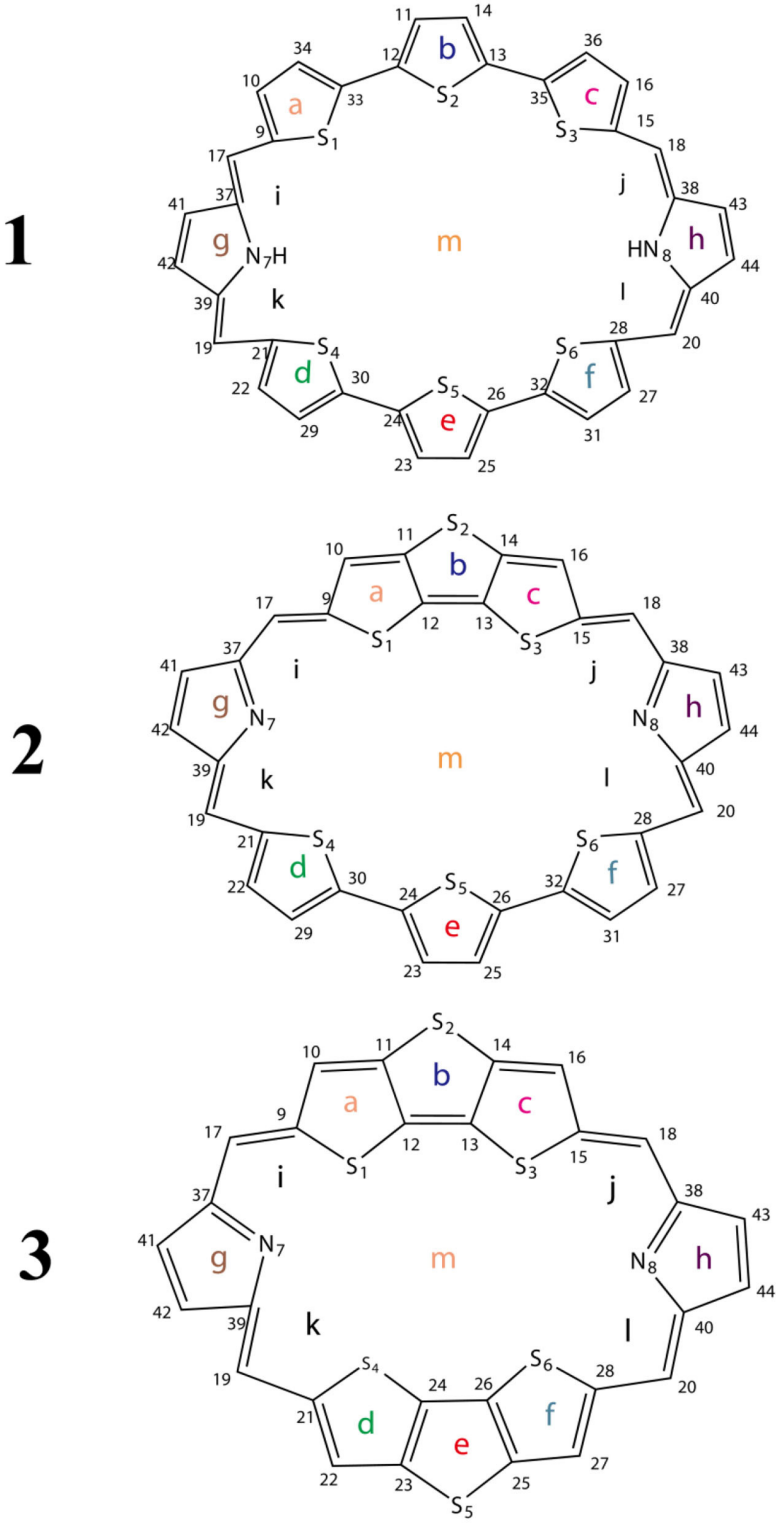
Sketch structures of molecules **1**–**3**.

In this work, we theoretically designed three macrocycle molecules **1**–**3** (Scheme 1) based on TTH and DTT. The aromaticity, stability, and photophysical properties of these molecules are investigated carefully using high-level quantum chemistry calculations. The potential high-efficiency macrocyclic molecules are screened. We hope these theoretical studies could pave the way for designing novel materials for a variety of applications.

## Computational Details

All calculations are performed using density functional theory (DFT) and time-dependent DFT (TD-DFT) methods as implemented in Gaussian 09 software package (Frisch et al., 2009). Full optimization of **1**–**3** was carried out using the B3LYP/6-311G (d, p) level of theory using DFT. Vibrational frequencies are calculated for the optimized structure at the same theory level to confirm the local minima. Vertical electronic excitations are calculated using TD-DFT at range-separated CAM-B3LYP functional together with 6-311G (d, p) basis set.

To avoid the in-plane components, the NICS values are calculated at 1 Å above the thiophene ring of DTT (points a, b, c, d, e, and f), pyrrole ring of TTH (points g and h), molecular center (point m), and intramolecular (points i, j, k, and l) using the flow around the gauge-independent atomic orbital method at the B3LYP/6-311G (d, p) level of theory. Illustration of the different critical points is detailed in Scheme 1. The critical points were analyzed by means of the *atoms in molecules* theory as implemented in the AIM2000 package (Bader et al., 1994).

## Results and Discussion

**Figure 1:**
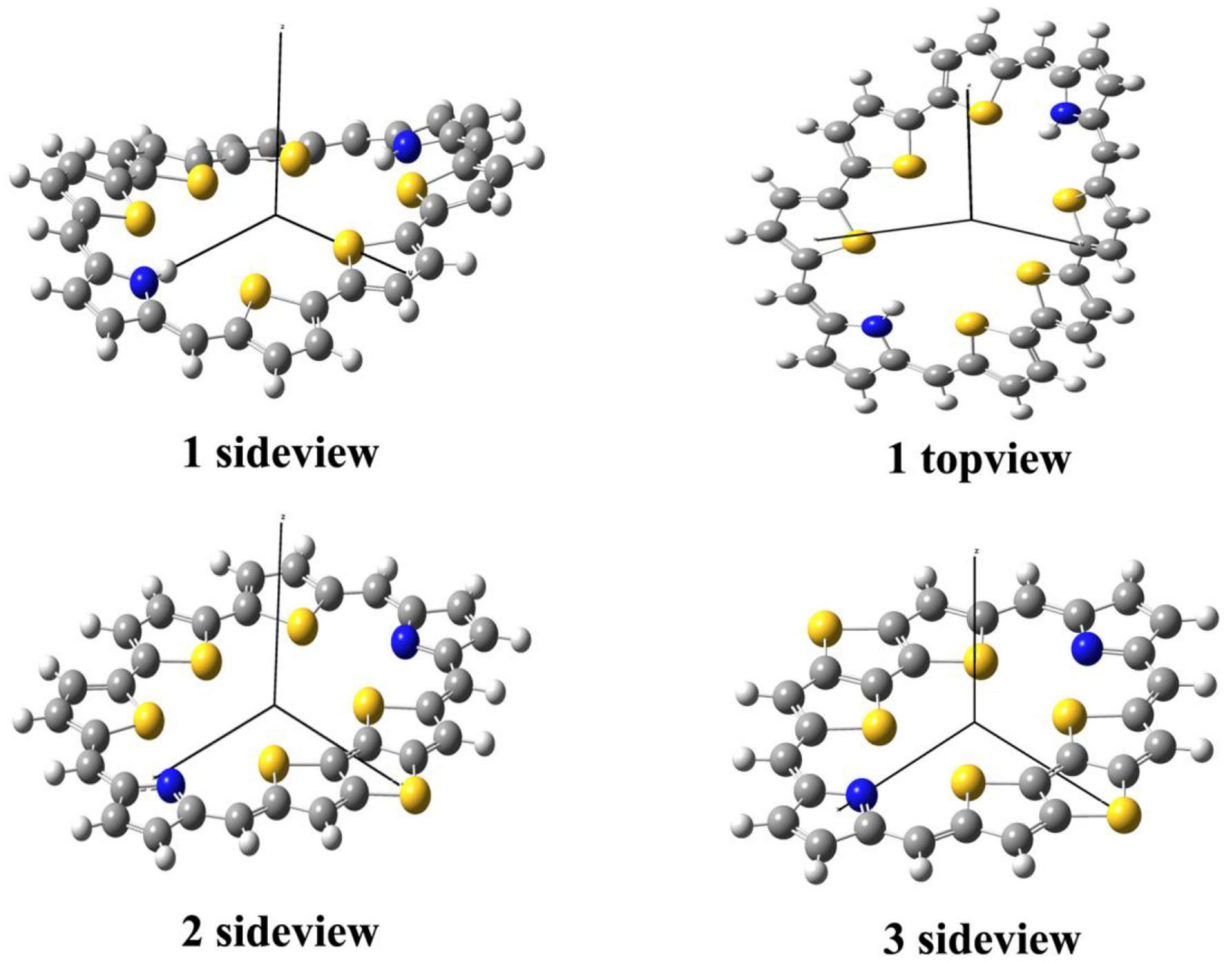
Optimized molecular structures of **1**–**3** in their ground states.

The optimized ground state structures of **1**–**3** are presented in Figure 1. Both TTH and DTT have three thiophene rings, and the difference is that TTH has the C-C single-bond bridge. For **1**, presence of two TTH units permutes the pyrrole group of the porphyrin ring, whereas for **2**, both TTH and DTT replace the pyrrole unit of the porphyrin ring. For **3**, two DTTs replace the pyrrole unit of the porphyrin ring. To quantitatively evaluate the structure difference of all molecules, we listed the selected geometrical parameters in Table 1. As can be seen from Table 1, the N7-N8 bond lengths of **1**, **2**, and **3** are 11.594, 9.820, and 8.351 Å, respectively. The C13–C26 bond lengths in **1**, **2**, and **3** are 8.065, 7.036, and 5.732 Å, respectively. It is apparently that N7–N8 and C13–C26 bond length decrease in the order of **1** < **2** < **3**. We also find similar observations for bond angles, which are 131.4, 131.0, and 126.0° (C9–C17–C37 bond angle) and 134.1,128.1, and 126.0° (C39–C19–C21 bond angle), respectively. It should be noted that the C9–C17 and C19–C21 bond lengths have the similar values of 1.4 Å and are rather intolerant to the use of either TTH or DTT units. From these data, we can conclude that macrocyclic rings get smaller from **1** to **2** and to **3**.

**Table 1:** Main optimized geometry structure parameters of **1**–**3**.

	**1**	**2**	**3**
**Bond lengths** **(Å)**			
N7-N8	11.594	9.820	8.351
C13-C26	8.065	7.036	5.732
C9-C17	1.394	1.391	1.396
C19-C21	1.394	1.398	1.399
**Bond angles (°)**			
C9-C17-C37	131.4	131.0	126.0
C39-C19-C21	134.1	128.1	126.0
**Dihedral angles (°)**			
C9-C17-C37-N7	5.6	0.0	0.0
C39-C19-C21-S4	−0.5	0.0	0.0
S5-C26-C32-S6	−13.1	0.0	-

**Scheme 2:**
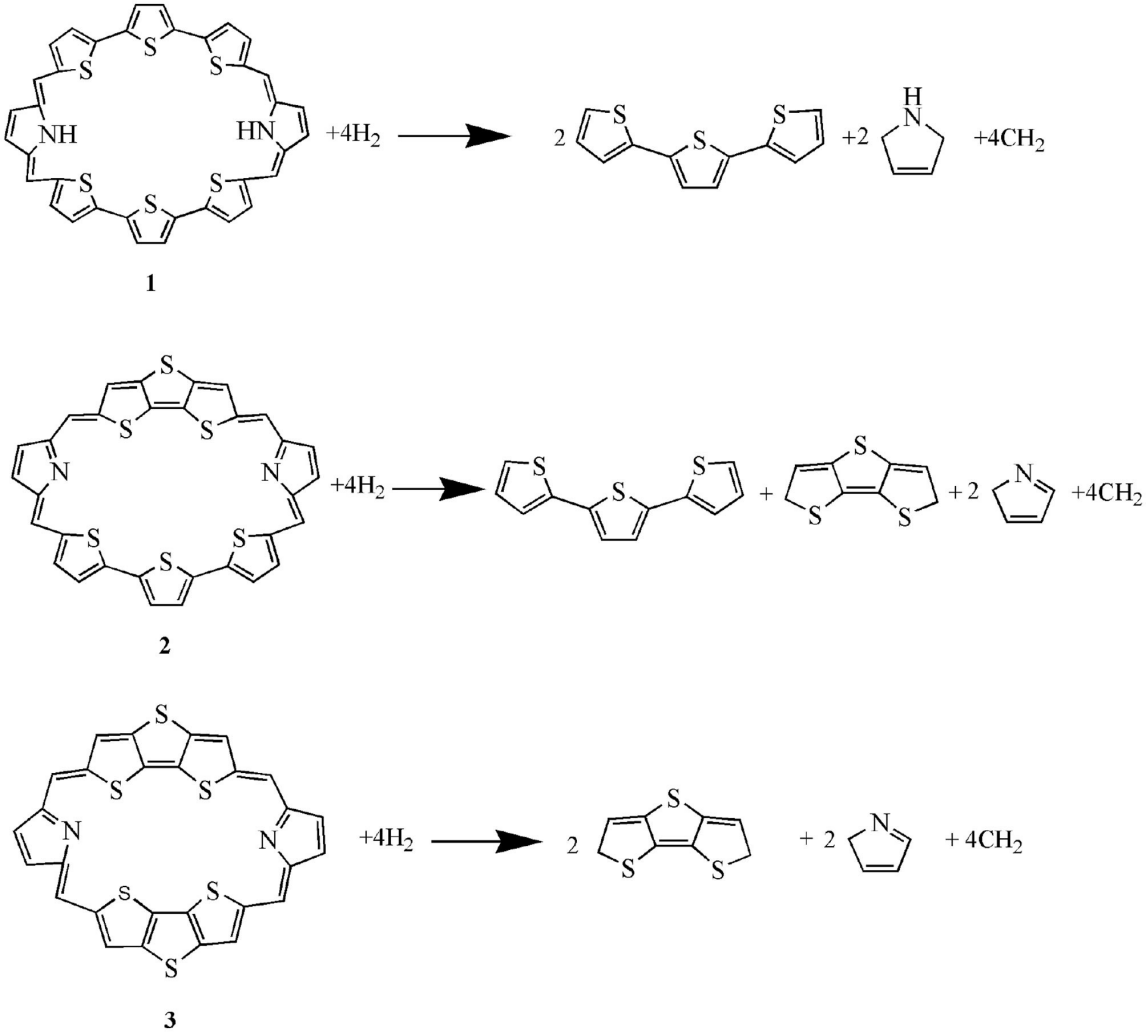
The isodesmic reactions of **1**–**3**.

The calculated dihedral angles of C9-C17-C37-N7, C39-C19-C21-S4, and S5-C26-C32-S6 of **1** are 5.6, 0.5, and 13.1°, respectively. Figure 1 shows that **1** adopts a propeller structure. In contrast, all dihedral angles in **2** and **3** are almost zero, indicating that **2** and **3** would have good coplanarity. Aromatic stabilization energy (ASE) is an important parameter for understanding stability and aromaticity from an energetic perspective (Yang et al., 2012). Here ASE values are calculated and listed in Table 2. We choose TTH, DTT, and 5-membered ring as the reference structures that present localized single and double bonds (see Scheme 2). Generally, the molecule with a smaller (more negative) ASE value (in absolute value) would have stronger stability. It is shown from Table 2 that the calculated ASE values decrease in the order of **1** > **2** > **3**, indicating that stability of molecules increases in an inverse order.

**Table 2:** Computed aromatic stabilization energy (ASE, in kcal/mol) of molecules **1**–**3**.

	**1**	**2**	**3**
ASE	−365.4	−387.3	−415.6

In order to gain deep understanding toward the stability, the aromaticity of atomic rings of all molecules is calculated. Research on aromaticity can help us to understand the stability essence of aromatic compounds (Aihara, 1999). More negative (smaller) NICS value denotes better aromaticity (Chang et al., 2005; Kirilchuk et al., 2017; Akaishi et al., 2018). The calculated results, as listed in Table 3, show that NICS (1) values at 1 Å above the critical points a, c, d, and f for **1**–**3** are similar. An exception is that the NICS (1) value of **1** at 1 Å above the critical point of ring b is more negative, whereas NICS (1) values of **2** and **3** at 1 Å above the critical point of ring b have smaller absolute values compared with **1**. It shows that the middle thiophene moiety in TTH has good aromaticity than in DTT. In addition, the NICS (1) values of **1** and **2** at 1 Å above the critical points of ring e have more negative values, whereas the **3** has a smaller negative value. It also indicates that the middle thiophene moiety in TTH has good aromaticity than in DTT. Moreover, the absolute NICS (1) values of **1**–**3** at 1 Å above the critical points of rings g and h are generally small and similar. The intramolecular 1 Å above critical points i, j, k, and l for **1**–**3** has the similar observation as 1 Å above critical points of rings g and h. As for the molecular center 1 Å above critical point, the NICS (1) values are −15.12, −15.56, and −16.23 ppm for **1**, **2**, and **3**, respectively, as listed in Table 3. It can be concluded from the abovementioned discussions that all molecules have strong aromaticity in the order of **1** < **2** < **3**. And the NICS value of porphyrin is −14.98 ppm (Wei et al., 2012). This also shows that **1**, **2**, and **3** have good aromaticity than porphyrin.

**Table 3:** NICS (1) values for **1**–**3**.

**NICS (ppm)**	**1**	**2**	**3**
a Thiophene ring NICS (1)	−23.82	−23.64	−22.19
b Thiophene ring NICS (1)	−24.82	−0.42	−0.53
c Thiophene ring NICS (1)	−23.82	−23.64	−22.19
d Thiophene ring NICS (1)	−23.82	−23.72	−22.19
e Thiophene ring NICS (1)	−24.82	−25.36	−0.56
f Thiophene ring NICS (1)	−23.82	−26.43	−22.19
g Pyrrole ring NICS (1)	−0.26	−0.16	−0.72
h Pyrrole ring NICS (1)	−0.26	−0.16	−0.72
i Intramolecular critical point NICS (1)	−23.76	−23.34	−23.12
j Intramolecular critical point NICS (1)	−23.76	−23.34	−23.12
k Intramolecular critical point NICS (1)	−23.76	−24.42	−23.12
l Intramolecular critical point NICS (1)	−23.76	−24.42	−23.12
m Molecular ring center NICS (1)	−15.12	−15.56	−16.23

**Figure 2:**
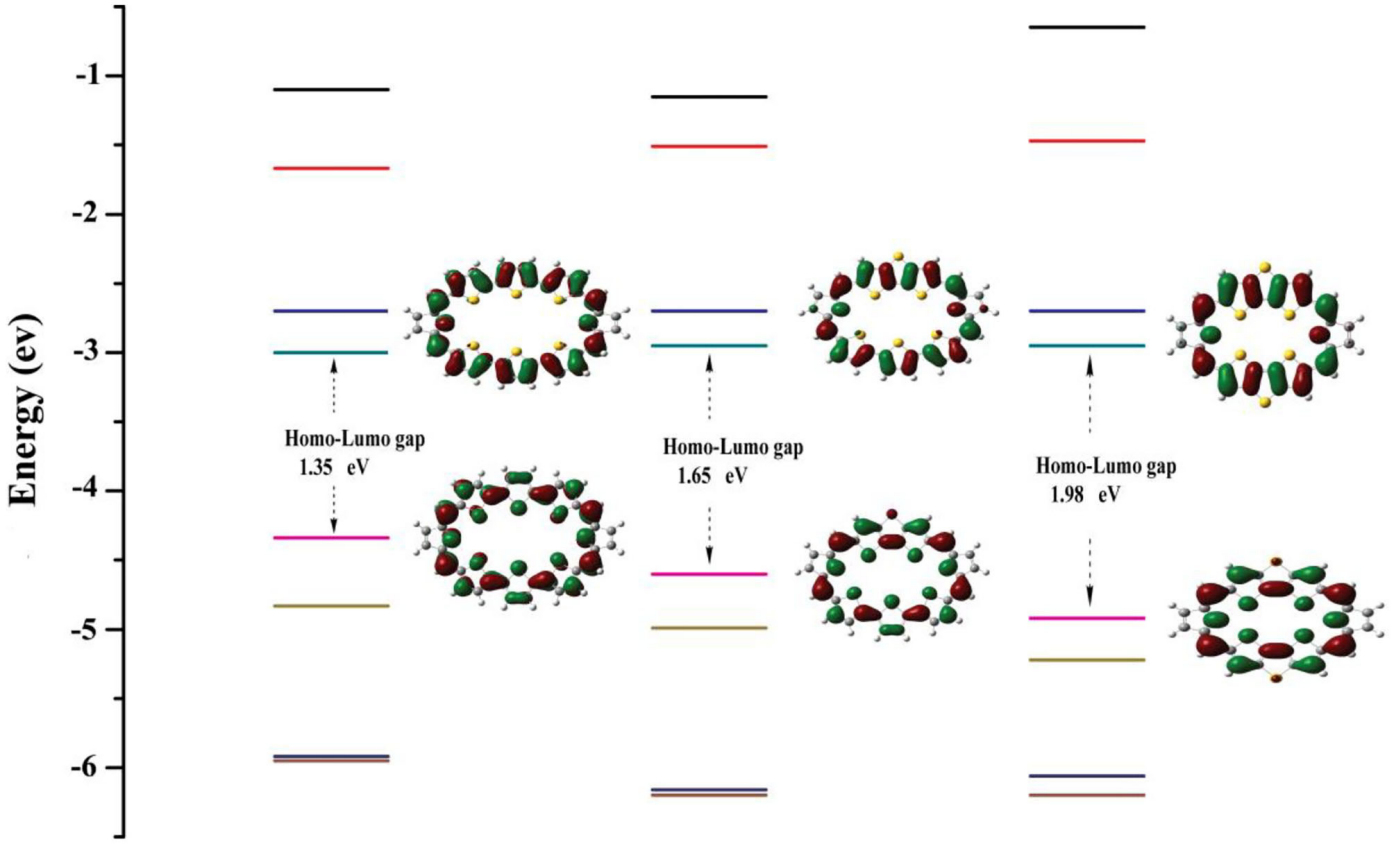
Molecular orbital distributions and diagrams of the frontier molecular orbitals (from HOMO-3 to LUMO+3) for **1**–**3**.

Energy levels of HOMO, LUMO, and HOMO–LUMO energy gap (Δ_H−L_) are meaningful to characterize optical and electronic properties. The frontier molecular orbital diagrams and Δ_H−L_ for **1–3** are shown in Figure 2. Inspection of Figure 2 reveals that, for all molecules, both HOMO and LUMO spread over the whole π-conjugated backbones. Specifically, HOMO is a π orbital and exhibits bonding, whereas the LUMO is a π^*^ orbital with antibonding character. This type of distribution is beneficial for intramolecular charge transfer process. A larger HOMO–LUMO energy gap would hamper the optical excitation, which induces better stability. The HOMO–LUMO energy gaps are 1.35, 1.65, and 1.98 eV for **1**, **2**, and **3**. Interestingly, the positive correlation between NICS (in absolute value) and HOMO–LUMO energy gaps can be observed, which is the molecule with the larger HOMO–LUMO gaps would have the larger absolute NICS value. This agrees well with previous work (Wei et al., 2019).

TD-DFT method is used to calculate the vertical electronic excitations. The simulated electronic configurations are reported in Table 4. From Table 4, it is obvious that the absorption features of all molecules are in the visible region. The major electronic excitations are from HOMO-1 → LUMO and HOMO → LUMO+1 transition, which is in contrast to typical HOMO → LUMO transitions of most porphyrin molecules. One can also observe that the absorptions are significantly red-shifted and broadened when incorporating TTH moiety. For **3**, the lowest-lying excitation is calculated to be 758 nm, and the major transition of HOMO → LUMO+1 has an oscillator strength of 0.0205.

**Table 4:** Calculated absorption features of molecules **1**–**3**.

**Molecule**	**E/nm (eV)**	**Major contribution**	**Oscillator strength**
**1**	1,041 (1.19)	HOMO-1 → LUMO (45%) HOMO → LUMO +1 (55%)	0.0041
**2**	892 (1.39)	HOMO-1 → LUMO (46%) HOMO → LUMO+1 (54%)	0.0087
**3**	758 (1.64)	HOMO-1 → LUMO (48%) HOMO → LUMO+1 (51%)	0.0205

**Figure 3:**
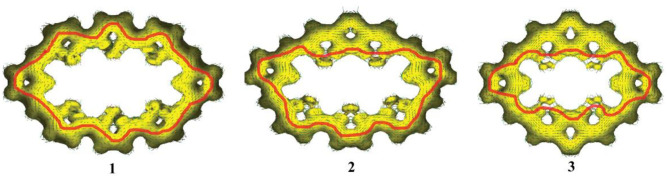
Computed AICD plots of **1**–**3** with an isosurface value of 0.03. Aromatic species exhibit clockwise diatropic circulations.

The aromaticity of all molecules is further confirmed by the anisotropy of the induced current density (AICD) analysis, which is a very popular method to investigate and quantify the delocalization in organic molecules (Herges and Geuenich, 2001; Geuenich et al., 2005)The AICD plots of **1**–**3**, with an isosurface value of 0.03, are shown in Figure 3. In general, aromatic species exhibit clockwise diatropic circulation, whereas antiaromatic compounds have paratropic circulations. The clockwise current density vectors plotted on the AICD isosurface confirm the presence of aromaticity in **1**–**3**. Figure 3 also shows that, for **1**, the current density flow locates around the outer section of rings a, b, and c of TTH. For **2**, the current density flow is around the inner section of rings a and b and rings a and c of DTT, while the current density flow is around the outer section of rings d, e, and f of TTH. For **3**, the current density flow is distributed around the inner section of rings a, b, and c of DTT. For **1**–**3**, the current path passes flow around the outer section of the two pyrrole rings.

On the other hand, for **1**, there is an important stabilizing interaction (second-order perturbation energy is about 2.32 kcal/mol) between the lone pairs of sulfur atom in the thiophene and the two closer *meso* π^*^ antibonding C-C. Small second-order perturbation energy results in the current density flow located around the outer section of TTH. For **3**, the stabilizing interaction of thiophene and two closer *meso* π^*^ antibonding C-C is much larger (about 25.48 kcal/mol), which enables current density flow distributed around the inner section of rings a, b, and c on DTT moiety. For **2**, the second-order perturbation energies between sulfur atom and the *meso* π^*^ antibonding C-C of TTH and DTT are 2.08 and 24.18 kcal/mol, rationalizing the involvement of different location (outer section of TTH vs. inner section of DTT) in current density flow distribution.

## Conclusion

In summary, we have investigated the stability, aromaticity, and photophysical properties of **1**–**3** with TTH and DTT units using DFT and TD-DFT methods. All molecules show high aromaticity and excellent photophysical properties. The calculation data suggest that the molecule with DTT is more stable than the one with TTH because of the better coplanarity in the former. The absorption features of the molecules are all located in the visible region. The major transitions for all molecules are from HOMO → LUMO+1 and HOMO-1 → LUMO, beneficial for intramolecular charge transfer process. Compared to molecules **1** and **2**, **3** stands out because of the increased HOMO–LUMO energy gap, more planar structure, and stronger aromaticity. Moreover, the current density flow for **3** is distributed around the inner section of DTT, in contrast to **1** in which the outer section of TTH dominates the current density flow; this arises from the different stabilizing interaction of thiophene and two closer *meso* π^*^ antibonding C-C, rationalizing the origin of different planarity, stability, and aromaticity. These theoretical results pave the way for future development of novel porphyrin molecules.

## Data Availability Statement

All datasets generated for this study are included in the article/supplementary material.

## Author Contributions

WW conducted the calculation and drafted the manuscript. WR, WJ, and BX assisted the calculation. HZ, F-QB, and WL proposed the idea and revised the manuscript. All authors contributed to the article and approved the submitted version.

## Conflict of Interest

The authors declare that the research was conducted in the absence of any commercial or financial relationships that could be construed as a potential conflict of interest.
